# Research funding impact and priority setting – advancing universal access and quality healthcare research in Malaysia

**DOI:** 10.1186/s12913-019-4072-7

**Published:** 2019-04-24

**Authors:** Weng Hong Fun, Sondi Sararaks, Ee Hong Tan, Kar Foong Tang, Diane Woei Quan Chong, Lee Lan Low, Roslinda Abu Sapian, S. Asmaliza Ismail, Suresh Kumar Govind, Siti Haniza Mahmud, Shahnaz Murad

**Affiliations:** 1 Institute for Health Systems Research, Ministry of Health Malaysia, Shah Alam, Selangor Malaysia; 2 National Institutes of Health Secretariat, Ministry of Health Malaysia, Shah Alam, Selangor Malaysia; 3 Department of Parasitology, Faculty of Medicine, University of Malaya, Kuala Lumpur, Malaysia; 4 Office of Deputy Director General of Health, Research and Technical Support, Ministry of Health Malaysia, Putrajaya, Malaysia

**Keywords:** Universal access, Quality healthcare, Payback framework, Health research priority setting, Research priority areas, Research impact, Stakeholder values, Stakeholder engagement, Research funding

## Abstract

**Background:**

Health Research Priority Setting (HRPS) in the Ministry of Health (MOH) Malaysia was initiated more than a decade ago to drive effort toward research for informed decision and policy-making. This study assessed the impact of funded prioritised research and identified research gaps to inform future priority setting initiatives for universal access and quality healthcare in Malaysia.

**Methods:**

Research impact of universal access and quality healthcare projects funded by the National Institutes of Health Malaysia were assessed based on the modified Payback Framework, addressing categories of informing policy, knowledge production, and benefits to health and health sector. For the HRPS process, the Child Health and Nutrition Research Initiative methodology was adapted and adopted, with the incorporation of stakeholder values using weights and monetary allocation survey. Workshop discussions and interviews with stakeholders and research groups were conducted to identify research gaps, with the use of conceptual frameworks to guide the search.

**Results:**

Seventeen ongoing and 50 completed projects were identified for research funding impact analysis. Overall, research fund allocation differed from stakeholders’ expectation. For research impact, 48 out of 50 completed projects (96.0%) contributed to some form of policy-making efforts. Almost all completed projects resulted in outputs that contributed to knowledge production and were expected to lead to health and health sector benefits. The HRPS process led to the identification of research priority areas that stemmed from ongoing and new issues identified for universal access and quality healthcare.

**Conclusion:**

The concerted efforts of evaluation of research funding impact, prioritisation, dissemination and policy-maker involvement were valuable for optimal health research resource utilisation in a resource constrained developing country. Embedding impact evaluation into a priority setting process and funding research based on national needs could facilitate health research investment to reach its potential.

**Electronic supplementary material:**

The online version of this article (10.1186/s12913-019-4072-7) contains supplementary material, which is available to authorized users.

## Background

Health research priority setting (HRPS) is essential to optimise the impact of health systems research investment [1], as the process of setting health research priorities can improve the efficiency of research fund utilisation and reduce duplication [2]. With this approach, a country could identify the health research needed in a transparent and systematic way [3], as well as align research initiatives to current needs [4].

Considerable investment goes into health research yearly, yet the resources required for health research funding far exceed availability. On top of this, Malaysia’s health system continues to face challenges in rising healthcare demands, shift in disease burden from infectious to non-communicable diseases, and in demographic transition [5]. Ultimately, the national health goal is to improve the health of Malaysia’s population by ensuring universal access and quality healthcare (UAQH), as highlighted in Malaysia’s strategic plans for national development (termed as Malaysia Plan (MP)) [6].

In Malaysia, the National Institutes of Health (NIH), a government agency with a network of research institutes under the Ministry of Health (MOH) Malaysia, funds health research initiatives that are aligned with national health priorities. Within the NIH and MOH, there have been ongoing efforts to address the gap between research, action and policy [7].

One of the key efforts was the establishment of HRPS in MOH. In 2011, the HRPS for the tenth MP had identified and prioritised health research areas in the following clusters: health systems, healthy lifestyles, empowerment, burden of disease, health technology and sustainable environment. Under health systems research, priorities were categorised into the domains of health financing and economics, governance, health information, human resources for health and service delivery [7]. These research domains were retained in the HRPS for the eleventh MP in 2017 and were collectively identified as the UAQH cluster [8].

Despite the establishment of HRPS processes in MOH, the impact and implications of research in supporting informed decision and policy-making are minimally known; only research output data such as the number of reports, publications and presentations produced were collected.

It is recognised that research impact assessment is challenging due to the lack of systematic approaches to evaluate research impact, particularly impact on health policy and practice [9]. Various frameworks have been developed to overcome this issue [10], such as the Payback Framework [11], UK Research Excellence Framework (REF 2014) [12], and the Becker Medical Library Model [13]. Among these models, the Payback Framework is one of the most commonly used frameworks [14]. Developed by the Health Economics Research Group (HERG), the framework defines research impacts into five categories [11], that they and others subsequently presented as: knowledge production, benefits to future research and research use, informing policy and product development, health and health sector benefits as well as broader economic benefits [15, 16]. The Payback Framework addresses conceptual issues and is applied to collect, analyse and report data consistently to capture research impacts and outputs, serving as a tool to assist funders and stakeholders to evaluate possible impact from research [17].

This study aimed to assess the impact of funded prioritised research to improve future HRPS and research fund allocation. We used the Payback Framework [11] to assess the impact of research projects, adopted the Child Health and Nutrition Research Initiative (CHNRI) methodology [1] for the identification and determination of research priority areas and used relevant conceptual frameworks for UAQH research gap identification.

## Methods

**Fig. 1:**
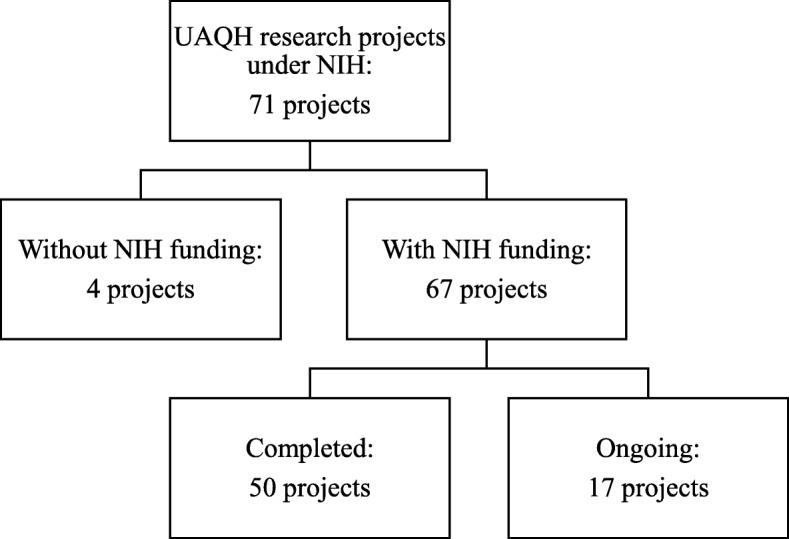
Flow chart of projects funded under Universal Access and Quality Healthcare Research, 10th Malaysia Plan

We assessed NIH-funded research projects in the 10th MP, from 2011 to 2015 [18], to inform priority setting for the 11th MP. Projects were included if the research objectives fulfilled research areas under the cluster of UAQH research [7]. A total of 71 projects were identified, and verified using administrative data from NIH and the National Medical Research Register database [19]. From these 71 projects, 67 were included and 4 excluded, as these did not receive direct NIH funding (Fig. 1).

### Evaluation of research funding and impact

Projects identified under the cluster of UAQH in the 10th MP were categorised according to the domains of the World Health Organization (WHO) framework [20]: Governance, Health Economics (HE), Human Resources for Health (HRH), Information & Technology (IT) and Service Delivery (SD). Distribution of research funds were analysed based on these domains.

#### Stakeholder survey

Stakeholders consisted of researchers, decision makers, healthcare providers, research funders and academicians, from government and private sectors. Stakeholders’ perspectives and expectations on each of the five domains’ importance were elicited in the form of percentages. These values were considered as the intended fund allocation. If all domains had equal importance, each would be allocated 20%. However, if some domains were more important than others, the percentage allocated to each domain would differ. The feedback scores from stakeholders were used to compare against the 10th MP NIH research fund allocation. In November 2016, approximately 200 questionnaires were distributed to all participants of the NIH Research Week and a research priority setting workshop, as well as emailed to 85 stakeholders who were invited to the workshop but could not attend. To maintain anonymity, participants were requested to drop off completed questionnaires into collection boxes available at the venue or to respond online.

#### Key informant interview

We conducted document review and multiple semi-structured interviews with the principal investigator and/or project team member(s) in a workshop setting for project impact assessment (Additional file 1). Out of the 67 projects that were reviewed, 50 were included for impact analysis as the remaining 17 were ongoing projects. The analysis focused on three impact categories, which were informing policy, knowledge production and benefits to health and health sector, adopting the modified Payback Framework by Kwan et al. [11, 21] and the Canadian Institutes of Health Research [9, 16, 22]. The impact category of benefits to health and health sector was set out to show benefits that could or were expected to result in improved service delivery, cost savings, improved health or increased equity [21].

Data on funds awarded to each project were obtained from NIH administrative records, and divided into tertiles [21], namely low (<RM32,600 (€6936)), moderate (RM32,601–87,000 (€6936-18,510)) and high (>RM87,000 (€18,510)), using the exchange rate in April 2017 of €1 = RM4.70 [23].

### Health research priority setting process

#### Identification of research gaps for the 11th Malaysia Plan

Health research priority areas of the 10th MP [7], national and global health plans [6, 18, 24, 25] were reviewed to identify national level research needs. We reviewed literature for conceptual frameworks to guide the identification of research gaps, and to increase comprehensiveness and inclusivity for each domain. Research gaps were grouped according to the domains of the WHO framework [20].

#### Research priority setting

We adopted and adapted the CHNRI methodology [1], a frequently used method to gain consensus on research areas. This involved setting criteria for prioritisation, incorporating stakeholders’ values through the application of weights, scoring research gaps, and calculating final scores for initial prioritisation. Stakeholders deliberated on this and identified the revised prioritised areas for the 11th MP.

#### Setting criteria for prioritisation

In setting criteria for prioritisation, we reviewed literature [1, 3, 26–28] and selected three (i.e. answerability/feasibility, importance/potential impact and magnitude/severity), in which each consisted of three questions (Additional file 2) chosen based on applicability, prior use in previous HRPS [7] and consensus from the project team. These criteria were applied in the stakeholders’ values instrument and research gaps assessment.

#### Incorporating stakeholders’ values

The stakeholder survey instrument contained a section on stakeholders’ values regarding prioritisation (Additional file 3). We incorporated these values as weights for the criteria, adopting the method of allocating monetary value [1, 3]. Stakeholders were asked to rate the importance of each criterion by allocating RM100 across the three criteria. For example, if all criteria had equal importance, each criterion should be allocated RM33.33. However, if one was more important, the allocation could be different. For example, the allocation could be as follows: answerability/feasibility criterion = RM65, importance/potential impact criterion = RM15 and magnitude/severity criterion = RM20. Regardless of research area, the total amount allocated to all criteria should add up to RM100.

#### Scoring of research gaps

In scoring each research area/gap, we asked the project team, predominantly researchers, to assess each area/gap by answering the questions for the criteria (see Additional file 2). We awarded 1 point to answers indicating agreement, 0.5 points for neither agreement nor disagreement, and 0 points for disagreement.

#### Calculation of scores for prioritisation

Summation of scores from the three criteria resulted in an intermediate score for each research area/gap. The stakeholders’ values in the form of monetary allocation for each criterion were incorporated as weighted mean score, representing the values of non-research expert groups. Thus, each research area’s final score consisted of the collective inputs of both researchers and stakeholders, similar to the method described by Rudan et al. [1] and was used for ranking. Further information on the calculation of weights to reflect stakeholders’ values can be found in Additional file 4.

### Statistical analysis

All statistical analyses were conducted using Statistical Package for the Social Science Version 21. The One-Sample Wilcoxon Signed Rank Test was conducted to test the association between stakeholders’ values for the three criteria and hypothetical values.

## Results

### Research funding and impact

Among the five research domains, differences exist in the distribution of research funds between the actual fund allocated and stakeholders’ expectations, with the lowest allocation for IT (4.6%) and the highest for SD (33.5%), likely due to the number of research projects in the respective domains (Table 1).

**Table 1:** Distribution of research fund allocation versus stakeholders’ expectations

Domain	Number of projects (*n* = 67)	Distribution of research funds^a^ (%)	
		10th MP fund allocation	Stakeholders’ expectations^b^
SD	29	33.5	25.7
HE	16	31.8	19.8
HRH	15	10.8	18.6
Governance	4	19.2	18.4
IT	3	4.6	17.4

^a^Percentages may not sum to 100% due to rounding. A total of 67 projects were analysed, including ongoing projects at time of analysis

^b^A total of 144 stakeholders responded

Response rate was 100% for key informant interview. Of the 50 completed projects, 36.0% (*n* = 18) supported decision and policy-making. Another 60.0% (*n* = 30) were reported to have led to policy-maker engagement and/or future agenda setting. Two projects in the lowest tertile reported no policy-maker engagement (Table 2).

**Table 2:** Fund awarded for projects by policy impact level

	Impact on informing policy^a^ (*n* = 50)		
	Research had no policy-maker engagement	Research led to policy-maker engagement and/or future agenda setting	Research supported decision and policy-making
	*n* (%)		
Fund awarded^**b**^			
Low	2 (100.0)	11 (36.7)	4 (22.2)
Medium	0	10 (33.3)	8 (44.4)
High	0	9 (30.0)	6 (33.3)
Total	2	30	18
Years from project completion (mean = 0.75, median = 0.5)			
0	0	17 (56.7)	8 (44.4)
1	2 (100.0)	9 (30.0)	7 (38.9)
2	0	2 (6.7)	1 (5.6)
3	0	2 (6.7)	2 (11.1)

^a^Percentages may not sum to 100% due to rounding. Of 67 projects, 17 ongoing projects were excluded

^b^Low<RM32,600 (€6936); Moderate = RM32,601–87,000 (€6936-18,510); High>RM87,000 (€18,510), using the exchange rate in April 2017 of €1 = RM4.70 [23]

As an indicator of knowledge production impact, there were 148 presentations, 33 reports, 22 research highlights and 8 publications produced. Evaluation of impact under the category of benefits to health and health sector showed that 36 projects expected to see an impact related to quality of care, while 17 expected health system delivery benefits (Table 3).

**Table 3:** Knowledge production and expected benefits to health and health sector

Category	Domain (number of projects)					
	Governance (*n* = 3)	HE (*n* = 6)	HRH (*n* = 13)	IT (*n* = 3)	SD (*n* = 25)	Total (*N* = 50)
Knowledge production						
Publication	0	1	2	0	5	8
Report	2	1	7	3	20	33
Research highlight^a^	1	0	7	0	14	22
Presentation	16	9	41	9	73	148
Benefits to health and health sector^b^						
Appropriateness of intervention	0	0	0	2	7	9
Quality of care	1	0	13	1	21	36
Health system delivery	2	0	0	1	14	17
Epidemiology	0	0	0	0	3	3
Cost and cost-effectiveness	0	6	0	0	2	8
Improved effectiveness of public health policy	1	0	0	1	1	3

^a^Research highlight: A 4-page summary of research evidence (key messages or research results), using plain language that aims to communicate with stakeholders and/or public

^b^Benefits that could or were expected to result in improved service delivery, cost savings, improved health, or increased equity. Each project could report one or more expected benefits

### Health research priority setting process

Out of 150 respondents in the stakeholder survey, 63.3% were researchers/academicians, 16.0% were decision makers and the remaining 20.7% were healthcare providers and others. Majority (92.0%) were from MOH, while the remaining were from public/private universities and other ministries. Most had more than 3 years of experience in their designated positions. Stakeholders placed higher values on answerability and importance than magnitude (Table 4).

**Table 4:** Stakeholders’ values on criteria for priority setting

	Mean (SD)	Median	Maximum	Minimum	25th Percentile	75th Percentile	*p*-value*
Criteria (*n* = 141)^a^							
Answerability	34.69 (15.16)	33.30	75.00	3.00	25.00	45.00	0.864
Importance	34.00 (13.69)	33.30	90.00	10.00	20.00	40.00	0.948
Magnitude	31.48 (12.31)	30.00	80.00	7.00	20.00	35.00	0.003

*One-Sample Wilcoxon Signed Rank Test. Statistical significance (*p* < 0.05) between criteria score and hypothetical value (33.3%)

^a^Respondents with missing data were excluded from analysis

#### Research gaps and priorities

The outcome of the HRPS process was a comprehensive list of research gaps for UAQH with topics ranked and prioritised. An example of this is in Additional file 5, and the complete prioritised areas for UAQH health research document is available elsewhere [8]. In general, the list of priority areas for each domain comprised of new and ongoing UAQH issues, research scope, research sub-domain, gaps and needs with the associated rationale. New research areas include transparency in health planning and delivery process, readiness/utilisation of IT as well as transparency and sustainability in health financing.

## Discussion

This study shed light into the impact of UAQH research funded in 2011–2015 in Malaysia. The concentration of funding in HE and SD domains was evident, likely due to the large number of priority areas identified in the HRPS for 10th MP in those domains. Almost all completed projects achieved some measure of policy impact and had outputs for knowledge production. For fund allocation, stakeholders deemed the criteria of answerability and importance to be more valuable than magnitude and affirmed that SD should have a larger share of the pie. The use of multipronged approach of conceptual frameworks, literature search, stakeholder engagement and research gaps review from previous research efforts led to a more comprehensive list of research areas for future funding.

Research funding is driven by many factors, such as the interests of research funders and stakeholders. Often, it is unguided by research priorities, causing most funded research to have little contribution to health systems and policy [29]. For example, in Mexico, most health research did not contribute to health policies as many projects were funded without priority setting [30]. The need to set priorities for health research for effective fund utilisation is crucial, in view of the potential for health systems research to contribute towards health system strengthening for universal health coverage and quality healthcare [31].

The funded amount was not a prominent factor in determining research evidence uptake for MOH policy-making, consistent with the results from Wooding et al. [15]. Most funded projects achieved some level of policy impact, suggesting that disseminated research on prioritised areas with policy-maker engagement could increase likelihood of research evidence integration into policy. Literature shows that despite significant lag time for translation into policy, policy-maker engagement, needs-led research and dissemination could contribute towards evidence uptake [9, 32–35]. However, we did not compare research impact of prioritised with non-prioritised research.

The involvement of a wide range of stakeholders in the HRPS process provided insight into the value stakeholders placed on UAQH research. Additionally, it fostered process legitimacy [3]. Research priorities that correspond to the needs of funders and those who could benefit from research outputs improves the overall credibility and potential health impact [36]. The HRPS process employed in MOH Malaysia provided a means for effective stakeholder engagement in research priority setting.

The fund allocation survey revealed stakeholders’ preference on research domains, although these differed from the actual fund awarded to each domain. This is in line with the notion that research funding practices are not only influenced by perceptions and research interests, but by other factors such as evidence needs, research gaps and prioritised areas [29, 37]. The crux is to achieve a balance between research domain importance and equitable research funding.

Research gaps and priority areas identified in the HRPS process were based upon issues in developing and sustaining efforts to improve UAQH in Malaysia. From 2011 to 2015, there was a heavier emphasis on research under SD and it continues to be of high importance, likely due to demand, number of research gaps, and conversely, reflective of capacity strengthening needs in other domains. For example, substantial research gaps continue to exist under the domain of IT as research initiatives are constrained by inadequate IT knowledge, skill, and capacity [38], as well as budget.

Although priority setting is extremely complex and challenging with the large number of competing research ideas for limited available funding [39], this initiative shows promising evidence that HRPS undertaken for national health research priorities in a developing country could foster better health system outcomes. The stepwise approach in our methodology was beneficial in generating priorities for distinct health research areas, compared with other priority setting processes in low or middle-income countries that addressed broad areas or specified populations/domains such as child health and mental health [4, 40]. It is hoped that this experience could prove beneficial for other developing countries.

However, research projects assessed included only those funded by NIH. Excluded were projects with grants from other sources, either local or international, or funded through operational budgets. Additionally, this study specifically assessed projects grouped under the UAQH research cluster, and hence findings may not be applicable for other MOH research funding initiatives such as funding for environmental health, non-communicable diseases or burden of disease [7]. We did not attempt to identify the economic or capacity building benefits of NIH-funded health research due to data unavailability, but it could form part of a future research for a more holistic measurement of research impact. Policy-maker engagement availability was studied; future exploration into degree of engagement and its benefits would be valuable for funders and applicants to increase impact.

Although response rate was 100% with key project leader involvement, the inherent subjectivity of self-reporting and timing of impact assessment could still affect results. However, Hanney et al. [32] noted that on average, self-reported data did not seem to over-emphasise impact. This study considered all projects equally, irrespective of methodological quality, project size and impact significance, which limits and complicates efforts to draw lessons from the overall impact assessment [32]. Despite this, research impact assessment is still essential to build evidence to demonstrate the return on investment of research funds.

We strived for stakeholder inclusivity, similar to other HRPS efforts [3, 36], but it proved to be a challenge. Resource intensive, time sensitive, contextual and cyclical in nature, research priority setting is difficult yet beneficial for a developing country. Future research could assess the degree of knowledge translation strategies employed, extent of incorporation of national priorities into research agenda and fund allocation. Additionally, evaluation of HRPS process and the extent of achievement of desired outcomes could inform future HRPS initiatives [36, 41].

## Conclusion

The concerted efforts of evaluation of research funding impact, prioritisation, dissemination and policy-maker involvement were valuable for optimal health research resource utilisation in a resource constrained developing country. The assessment of research impact provided evidence to inform future priority setting, and the combination of priority setting approach with stakeholder engagement likely contributed to research impact. Despite modest funding and early phase of post-research completion, a fairly good level of benefit was noted for decision and policy making, with health and health sector gains expected, especially in quality of care and health system delivery. It is undeniable that embedding impact evaluation into a priority setting process and funding research based on national needs would facilitate health research investment to reach its potential.

## Additional files

Additional file 1: Key informant interview form. This form was used in the key informant interview. (DOCX 14 kb)

Additional file 2: Items within each criterion used for priority setting. This table describes the three criteria and their associated questions chosen to evaluate health research areas identified in the HRPS process. (DOCX 21 kb)

Additional file 3: Survey on Health Research Priority Setting: Values & Importance. This survey form was developed to elicit stakeholders’ values on research criteria and importance of each research domain. (DOCX 230 kb)

Additional file 4: Example of weight assigned for each criterion. This table describes the method used to calculate the average weight for each criterion, that takes into consideration the scores allocated by all stakeholders involved. (DOCX 18 kb)

Additional file 5: Examples of research priority areas and research gaps for UAQH in Malaysia. This table shows examples of UAQH research priority areas across five WHO domains and the associated research gaps and expected outcomes that were identified in the HRPS for 11th MP. (DOCX 17 kb)

## Abbreviations

CHNRI: Child Health and Nutrition Research Initiative
HE: Health Economics
HRH: Human Resources for Health
HRPS: Health Research Priority Setting
IT: Information and Technology
MOH: Ministry of Health Malaysia
MP: Malaysia Plan
NIH: National Institutes of Health
SD: Service Delivery
UAQH: Universal Access and Quality Healthcare
WHO: World Health Organization
