# Supplementary material for: Research funding impact and priority setting – advancing universal access and quality healthcare research in Malaysia
Source: BMC Health Serv Res. 2019 Apr 24;19:248. doi: 10.1186/s12913-019-4072-7 (PMC6480746; doi:10.1186/s12913-019-4072-7)
Supplement: Supplementary file 1 — Key informant interview form. This form was used in the key informant interview. (DOCX 14 kb) [file 12913_2019_4072_MOESM1_ESM.docx]

| **Additional file 1: Key Informant Interview Form** | | | | | | | | | |  |
| --- | --- | --- | --- | --- | --- | --- | --- | --- | --- | --- |
| 1 | Institution of Principal Investigator |  | | | | | | | |  |
| 2 | Theme (National Problem/UAQH issues) |  | | | | | | | |  |
| 3 | Category (Research Scope) |  | | | | | | | |  |
| 4 | Subcategory (Suggested Research Area) |  | | | | | | | |  |
| 5 | Fund Awarded |  | | | | | | | |  |
| 6 | Research Title |  | | | | | | | |  |
| 7 | Research Objectives |  | | | | | | | |  |
| 8 | Expected Output (Please list expected research project findings that can address national problem/UAQH issues) |  | | | | | | | |  |
| 9 | Expected benefit to health & health sector (Please choose one or more from category a-f) * |  | | | | | | | |  |
| 10 | Outputs (List of publication, report, research highlight, presentation etc) |  | Please provide title(s) and date of publication | | | | | | |  |
|  |  | Publication(s) |  | | | | | | |  |
|  |  | Report(s) |  | | | | | | |  |
|  |  | Research Highlight(s) |  | | | | | | |  |
|  |  | Presentation(s) |  | | | | | | |  |
|  |  | Objective 1 | Objective 2 | Objective 3 | | Objective 4 | | | Objective 5 |  |
| 11 | Research Objectives |  |  |  | |  | | |  |  |
| 12 | Key Findings |  |  |  | |  | | |  |  |
| 13 | Does the findings answer the expected output? (Yes or No) |  |  |  | |  | | |  |  |
| 14 | Based on question 13: If yes, how? If no, why? |  |  |  | |  | | |  |  |
| 15 | Overall impact of project on policy/decision-making (Please choose Level 1-3) **. Provide supporting evidence such as action taken to use research project findings in policy/decision-making, policy-maker engagement activities or which divisions in Ministry of Health were involved. |  | | | | | | | |  |
| 16 | Further action? (Other identified gaps to be addressed) |  | | | | | | | |  |
|  |  |  |  | |  | |  |  | |  |
| * | **Expected benefit to health and health sector** | | | |  | |  |  | |  |
| a | Appropriateness of intervention | | | |  | |  |  | |  |
| b | Quality of care | | | |  | |  |  | |  |
| c | Health system delivery | | | |  | |  |  | |  |
| d | Epidemiology | | | |  | |  |  | |  |
| e | Cost and cost-effectiveness | | | |  | |  |  | |  |
| f | Improved effectiveness of public health policy | | | |  | |  |  | |  |
|  |  |  |  | |  | |  |  | |  |
| ** | **Impact level** | | | |  | |  |  | |  |
| Level 1 | Research had no policy-maker engagement | | | |  | |  |  | |  |
| Level 2 | Research led to policy-maker engagement and/or future agenda setting | | | |  | |  |  | |  |
| Level 3 | Research supported decision and policy-making | | | |  | |  |  | |  |
