# Supplementary material for: Research funding impact and priority setting – advancing universal access and quality healthcare research in Malaysia
Source: BMC Health Serv Res. 2019 Apr 24;19:248. doi: 10.1186/s12913-019-4072-7 (PMC6480746; doi:10.1186/s12913-019-4072-7)
Supplement: Supplementary file 2 — Items within each criterion used for priority setting. This table describes the three criteria and their associated questions chosen to evaluate health research areas identified in the HRPS process. (DOCX 21 kb) [file 12913_2019_4072_MOESM2_ESM.docx]

Additional file 2: Items within each criterion used for priority setting.

| Criteria | Items |
| --- | --- |
| Answerability/feasibility | 1. Can a study be designed to provide a practical solution/outcome? |
|  | 1. Is it likely that, in the context of interest, there will be sufficient capacity to conduct this study in 11^th^ MP? |
|  | 1. Is the cost and time required for this study reasonable within the 11^th^ MP (2016-2020)? |
| Importance/potential impact | 1. Will the results of this research fill an important knowledge gap in achieving national goals (11^th^ MP)? |
|  | 1. Are the results from this research likely to shape future planning and implementation? |
|  | 1. Are the results of the research likely to be beneficial (equitable) to the community/society? |
| Magnitude/severity | 1. Is the problem common in terms of burden to the healthcare system/community? |
|  | 1. Is the problem severe to the healthcare system/community? |
|  | 1. Is data urgently needed for decision making? |
