# Supplementary material for: Research funding impact and priority setting – advancing universal access and quality healthcare research in Malaysia
Source: BMC Health Serv Res. 2019 Apr 24;19:248. doi: 10.1186/s12913-019-4072-7 (PMC6480746; doi:10.1186/s12913-019-4072-7)
Supplement: Supplementary file 3 — Survey on Health Research Priority Setting: Values & Importance. This survey form was developed to elicit stakeholders’ values on research criteria and importance of each research domain. (DOCX 230 kb) [file 12913_2019_4072_MOESM3_ESM.docx]

Survey on Health Research Priority Setting:

Values & Importance

| This will only take **5 minutes** of your time.  National Institutes of Health, MOH Malaysia is in the process of identifying priorities for health research. As part of the process, this survey seeks to identify the value of stakeholders as input to the priority setting for **Health Service and Management Cluster.**  There are two main sections:  - Value of criteria  - Importance of domains  We appreciate your feedback. Thank you. | | |
| --- | --- | --- |
|  | | |
| 1. How would you best describe yourself while answering this survey? (Mark only one) | | |
|  | Funder | |
|  | Healthcare Decision Maker/ Policymaker | |
|  | Researcher | |
|  | Other: ____________________________________ | |
| 1. How many years of experience do you have in this position (referring to the answer above)?   _________________________________________________________ | | |
| 1. Which institution are you mainly affiliated with? (Mark only one) | | |
| Ministry of Health | | Public University [state: ________________] |
| Other Ministry [state: ________________] | | Private University [state: ________________] |
| None | | Other: ___________________ |
|  | |  |
| **Scoring for Criteria** | | |
| There are 3 criteria. We need your help in identifying the value for each criterion, whether they are equal or more important than the other. These values will be used, as input (as weights) to rank research areas.  Each criterion is represented by 3 questions.  For each criterion, please rate the importance of the criterion to you. We have used the concept of **monetary allocation**, where the amount allocated to a criterion reflects its importance.  Total amount allocated to all 3 should add up to RM100.  For example, if all criteria are equally important, each will be allocated RM 33.33. If one is more important, the allocation could be, e.g. Criterion 1: RM65, Criterion 2:RM15, Criterion 3: RM20. | | |

| **Answerability/Feasibility means:**   - Can a study(ies) be designed to provide a practical solution/outcome? - Is it likely that, in the context of interest, there will be sufficient capacity to carry out in 11^th^ MP? - Is the cost and time required for this research reasonable within 11^th^ MP (2016-2020)? | | **Importance/Potential Impact means:**   - Will the result of this research fill an important knowledge gap in achieving national goals? - Are the results from this research likely to shape future planning and implementation? - Are the results of the research likely to be beneficial (equitable) to community/society? | | | **Magnitude/Severity means:**   - Is the problem common in terms of burden to the healthcare system/ community? - Is the problem severe to the healthcare system/ community? - Is data urgently needed for decision-making? | |
| --- | --- | --- | --- | --- | --- | --- |
| For example  **RM: 65** | | For example  **RM: 15** | | | For example  **RM: 20** | |
| Your answer  **RM: __________________** | | Your answer  **RM: ____________________** | | | Your answer  **RM: __________________** | |
| **Total: RM 100** | | | | | | |
|  | | | | | | |
| **Scoring of Research Domains** | | | | | | |
| There are 5 domains under the cluster of Health Services and Health Management.  We would like you to rate the importance of these domains. Please assign the % for each domain and the % allocated should reflect its importance.  Example, if all research domains have equal importance, 20% will be allocated to each. If one is more important, the allocation could be: Domain 1: 15%, Domain 2: 30%, Domain 3: 15%, Domain 4: 20% and Domain 5: 20%. Total amount allocated to all 5 should add up to 100%. | | | | | | |
| Domains | | | | | | |
| 1. Governance | 2. Health Economics | | 3. Information & Technology | 4. Health Service Delivery | | 5. Human Resources for Health |
| _______________% | _______________% | | _______________% | _______________% | | _______________% |
| **Total: 100%** | | | | | | |
| **Contact Details** | | | | | | |
| Please provide your email address for us to send you a copy of the results. | | | | | | |
| Email: _____________________________________________ | | | | | | |
| We welcome any comments and suggestions.  ___________________________________________________________________________________________  ___________________________________________________________________________________________ | | | | | | |
| **Please drop this into any HSHM values & importance survey box located at registration counter or HSHM Dialogue Room (Crystal Room).** | | | | | | |
