# Supplementary material for: Research funding impact and priority setting – advancing universal access and quality healthcare research in Malaysia
Source: BMC Health Serv Res. 2019 Apr 24;19:248. doi: 10.1186/s12913-019-4072-7 (PMC6480746; doi:10.1186/s12913-019-4072-7)
Supplement: Supplementary file 4 — Example of weight assigned for each criterion. This table describes the method used to calculate the average weight for each criterion, that takes into consideration the scores allocated by all stakeholders involved. (DOCX 18 kb) [file 12913_2019_4072_MOESM4_ESM.docx]

Additional file 3: Example of weight assigned for each criterion.

| **Criterion** | **Stakeholder A** | **Stakeholder B** | **Stakeholder C** | **Average weight for each criterion** |
| --- | --- | --- | --- | --- |
| Answerability/ feasibility | 20 | 25 | 40 | $(\frac{20+25+40}{3}$)/100  = 0.283 |
| Importance/ potential impact | 30 | 30 | 30 | $(\frac{30+30+30}{3}$)/100  = 0.300 |
| Magnitude/ severity | 50 | 45 | 30 | $(\frac{50+45+30}{3}$)/100  = 0.417 |
