# Supplementary material for: Research funding impact and priority setting – advancing universal access and quality healthcare research in Malaysia
Source: BMC Health Serv Res. 2019 Apr 24;19:248. doi: 10.1186/s12913-019-4072-7 (PMC6480746; doi:10.1186/s12913-019-4072-7)
Supplement: Supplementary file 5 — Examples of research priority areas and research gaps for UAQH in Malaysia. This table shows examples of UAQH research priority areas across five WHO domains and the associated research gaps and expected outcomes that were identified in the HRPS for 11th MP. (DOCX 17 kb) [file 12913_2019_4072_MOESM5_ESM.docx]

**Additional File 4: Examples of research priority areas and research gaps for universal access and quality healthcare in Malaysia.**

| **Domain** | **Code** | **Research Scope** | **Sub-domain** | **Gaps & Needs (Rationale)** | **Suggested Research Areas** | **Expected Output** |
| --- | --- | --- | --- | --- | --- | --- |
| Governance | GT17_01 | Health planning | Transparency | Lack of transparency is associated with poor participation of public in healthcare. This could inhibit citizens' efforts/ in giving voice to public, to hold health systems accountable.  Lack of transparency of health planning and delivery process as well as factors contributing to the accountability of healthcare providers. | Transparency in health planning and delivery process to promote and ensure the participation of public in healthcare planning and delivery. | Strategies/approaches that foster system design to promote transparency. |
|  | GT17_02 | Health regulation | Rules of law/control of corruption | Lack of assessment and identification of organisational factors related to corruption such as ghost workers, absenteeism or selling of public-funded resources e.g. drugs. | Occurrence of ghost workers, absenteeism etc. and inadequacies in organisational environment/ structure related to this such as supervision, monitoring mechanism etc. | Approach, intervention or policy change that addresses the inadequacies in organisations to alleviate the situation. |
| Human Resources for Health | RT17_01 | Human Resource Management (HRM) workforce lifespan | Performance Management: Productivity/ Retention and Exit | The outcomes of the programme/policies introduced for Human Resources for Health (HRH) benefit were not comprehensively assessed. | The effectiveness of strategies employed such as Full Paying Patient Programme (FPP), flexi system and other approaches. | Effectiveness and sustainability of current strategies in HRH management. |
|  | RT17_02 | HRM workforce lifespan | Work environment | Lack of comprehensive data on mental health status of HRH and practice environment. | Mental health status among HRH and practice environment, strategies to improve organisational effectiveness. | Strategies to improve mental health and practice environment. |
| Health Information & Technology | TT17_01 | Data | Data Interoperability (content) | Information exchange from health information systems do not happen directly without needing to map metadata, which affects the content of data collected. | The consequences, benefits and impact of data interoperability. | Strategies/guidelines for data to be used across different systems, without any change in meaning and function. |
| Health Economics | ET17_01 | Collection of resources | Resource generation | Lack of legal and regulatory framework, accountability and transparency.  Minimal public-private partnership.  Unclear about the sustainability of resource generation. | Private-public partnership in term of governance, resource generation and resource optimisation. | Formulation of legal and regulatory framework between public and private sector.  The best practice to enhance Private-Public Partnership. |
|  | ET17_02 | Distribution and utilisation of resources | Equity | Maldistribution of resources (money, manpower, asset and facility) at macro-level, state-level, programme level and between different providers (public or private). | Resource allocation based on health needs.   Measuring and standardising health needs at individual and population level.  Equity in distribution of human resources.  Equity in distribution between private and public health provider. | Framework/methodology/mechanism for macro-level resource allocation based on health needs  Guideline for equitable resource allocation.  Public and private sector roles in achieving Universal Health Coverage. |
| Service Delivery | ST17_01 | Quality of care | Person-centred care | Inadequate analysis, assessment and/or application of mortality, morbidity, public health (e.g. vaccination, occupational health, environmental hazards, etc.) as well as Traditional and Complementary Medicine (T&CM) practices and services and clinical data at practice or primary care level to identify and tailor programmes or services needed for the communities. | Community-oriented provision of health services at practice or primary care level, based on available data (e.g. clinical, mortality, morbidity, public health data, etc.).  To analyse and develop suitable assessment tools for T&CM practices and services, to have more evidence-based T&CM practices. | Strategies that enable or train healthcare innovators to conceptualise, implement, evaluate and disseminate community-oriented primary care programmes.  Develop tailor-made T&CM programme according to the community’s needs. |
|  | ST17_02 | Quality of care | Effectiveness and efficiency | Lack of evaluation of healthcare programmes, services, work processes including standard operating procedures in various areas (e.g. in child and maternal health, food and safety, laboratory, dental, pharmacy, T&CM and other levels of care or relevant fields).  Public and private healthcare facilities practice different approach of delivering T&CM services to the public. | Effectiveness, efficiency and health outcomes of programmes and services delivered. Examples include LEAN management of healthcare to improve effectiveness and efficiency; performance benchmarking; T&CM services in T&CM units in public hospital/ private healthcare facilities. | Provide inputs for the strengthening and revision of existing programmes, services, work processes including standard operating procedure, clinical practice guidelines and others. |
|  | ST17_13 | Access | Accessibility | Limited oral health services for toddlers, adults, population with special needs and vulnerable/ marginalised groups (e.g. the elderly and indigenous populations of Sabah and Sarawak). | Evaluation of oral health services, focusing on accessibility and utilisation of services by toddlers, adults, population with special needs and vulnerable/ marginalised groups; looking at current services and barriers such as waiting time, impact and manpower needs.  Assess the accessibility and affordability of oral healthcare services from users' perspective and public willingness to pay for these services. | Information for the improvement and expansion of oral health services. |
